# Association of Cardiac Sarcoidosis and Ulcerative Colitis: A Case Report and a Brief Review of the Literature

**DOI:** 10.1016/j.case.2024.12.010

**Published:** 2025-02-22

**Authors:** Alessio Cianci, Maria Chiara Meucci, Lucia Leccisotti, Stefano Bianchi, Qianqian Zhang, Francesca Graziani, Francesco Burzotta

**Affiliations:** a Department of Cardiovascular and Pneumological Sciences, Catholic University of the Sacred Heart, Rome, Italy; b Department of Cardiovascular Medicine, Fondazione Policlinico Universitario A. Gemelli IRCCS, Rome, Italy; c Section of Nuclear Medicine, Department of Radiological Sciences and Haematology, Università Cattolica del Sacro Cuore, Rome, Italy; d Unit of Nuclear Medicine, Fondazione Policlinico Universitario A. Gemelli IRCCS, Rome, Italy; e Arrhythmology Unit, Fatebenefratelli Isola Tiberina-Gemelli Isola Hospital, Rome, Italy; f Division of Anatomic Pathology and Histology, Fondazione, Policlinico Universitario A. Gemelli IRCCS, Università Cattolica del Sacro Cuore, Rome, Italy

**Keywords:** Cardiac sarcoidosis, Ulcerative colitis, Autoimmune disorders, T helper 17

## Abstract

• CS is a rare inflammatory disease with immune-mediated pathogenesis.
• The authors report a rare case of coexistence of CS and ulcerative colitis.
• CS and ulcerative colitis may share common pathogenetic pathways.
• A high level of suspicion for CS should be kept in patients with immune diseases.

## Graphical abstract

**Figure:**
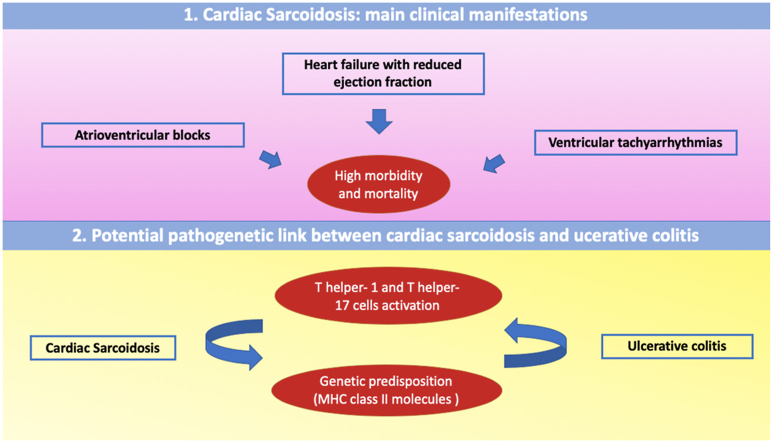

## Introduction

Sarcoidosis is a systemic disease characterized by a chronic immune-mediated inflammatory status leading to non-necrotizing granulomas formation, which may theoretically affect any organ, including the heart. Importantly, cardiac involvement is one of the main prognostic determinants in these patients.^1^^,^^2^ Ulcerative colitis is a chronic inflammatory disorder of the colon, with an immune-mediated pathogenesis.^3^ Here, we report a clinical case of association between ulcerative colitis and cardiac sarcoidosis. We discuss the potential pathogenetic links between the two diseases and the importance of an early diagnosis of cardiac sarcoidosis to prevent the evolution toward life-threatening complications.

## Case Presentation

A 52-year-old woman was referred to our center for a specialist cardiologic evaluation.

Three years before, the patient experienced a third-degree atrioventricular (AV) block, which required an urgent dual-chamber pacemaker implantation at another center. At that time, the patient underwent echocardiography, which showed preserved left ventricular (LV) systolic global function with LV wall thickening (mid and basal interventricular septal thickness 15 mm), and cardiac computed tomography (CT), which excluded obstructive coronary artery disease.

The patient had a history of hidradenitis suppurativa and ulcerative colitis, which was diagnosed 8 years before the hospitalization for AV block and treated with prednisone and mesalazine in the acute phase. However, the immunosuppressive treatment was spontaneously interrupted 2 years later, without clinical relapse.

Approximately 2 years later, the patient was admitted to the hospital because of a sustained ventricular tachycardia and underwent a subcutaneous implantable cardioverter-defibrillator (S-ICD) implantation. Echocardiography revealed thinning and akinesia of the basal interventricular septum (IVS) and normal LV ejection fraction (LVEF). These echocardiographic findings, along with the clinical picture, raised the suspicion of cardiac sarcoidosis. Cardiovascular magnetic resonance was not performed, because the previously implanted pacemaker was not magnetic resonance compatible and there were abandoned, retained leads in place from a previous revision, although this is no longer an absolute contraindication.^4^

**Figure 1:**
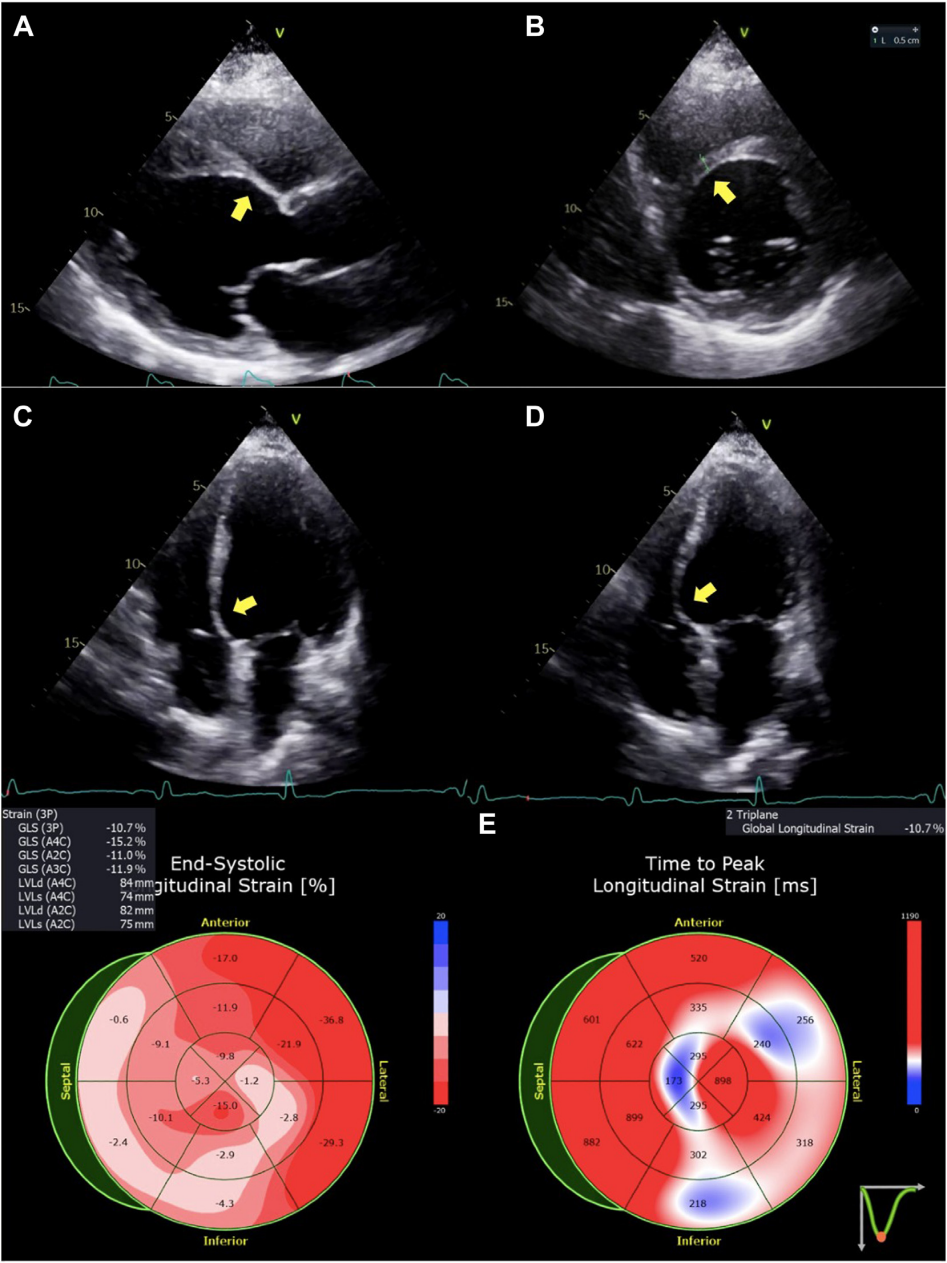
Two-dimensional transthoracic echocardiography, parasternal long-axis **(A)**, parasternal short-axis **(B)** diastolic, apical four-chamber diastolic **(C)** and systolic **(D)** views, demonstrates a dilated, hypokinetic LV cavity with regional myocardial thinning and scarring of the basal segment of the IVS with aneurysmal motion *(arrows)*; the bull’s-eye display **(E)** of the end-systolic *(left)* and time to peak *(right)* LV global longitudinal strain patterns demonstrates significantly reduced values with greater regional impairment of basal segments of the IVS and inferior walls.

A few months later, the patient was referred to our center, reporting shortness of breath with mild effort (New York Heart Association functional class III) and sporadic episodes of dizziness after S-ICD implantation. Transthoracic echocardiography demonstrated dilated cardiomyopathy with moderate to severe LV systolic dysfunction (LVEF 30%, biplane Simpson method), increased wall thickness in the mid IVS, and basal inferior and inferoseptal thinning and akinesia (Figure 1, Video 1, Video 2, Video 3, Video 4).

**Figure 2:**
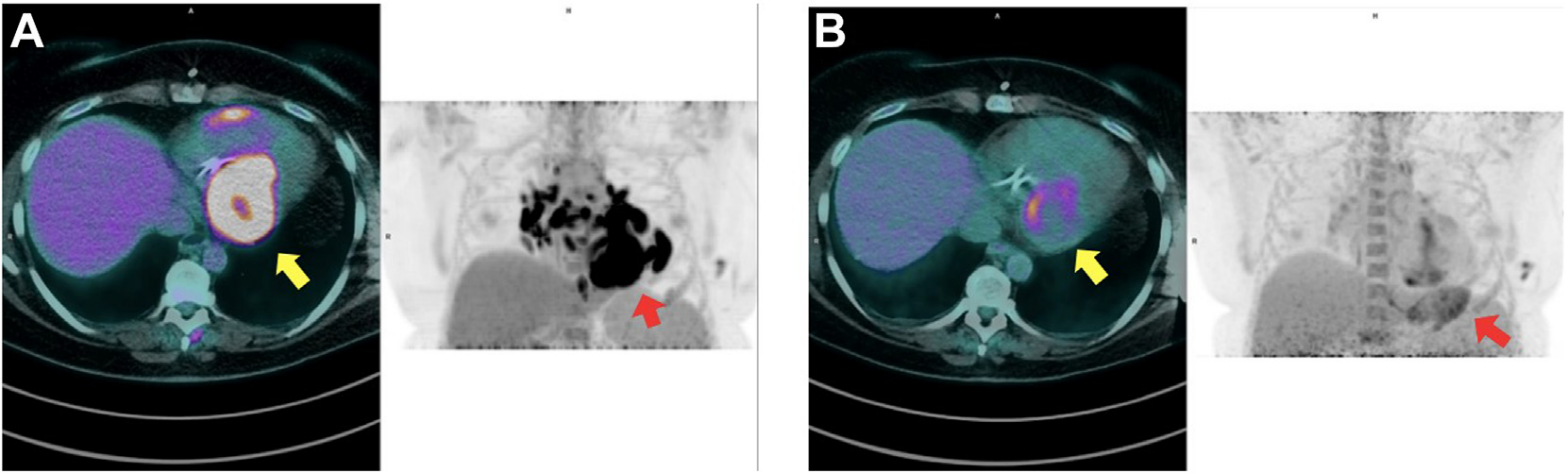
PET/CT at baseline **(A)** and at 4-month follow-up after immunosuppressive treatment **(B)** using 2-[^18^F]-fluorodeoxyglucose, axial displays at the level of the heart *(left, color figures)* and coronal whole-body displays *(right, black and white figures)*. Panel **A** demonstrates diffuse and patchy metabolic activity particularly involving the IVS, inferior LV wall, and right ventricular free wall, as well as multiple foci of intense metabolic activity involving mediastinal lymph nodes and less intense activity in pulmonary nodules and the hilum; panel **B** shows partial metabolic response in the heart with minimal residual metabolic activity in the inferior basal LV wall (maximum standardized uptake value 7 vs 18) and complete metabolic response of the mediastinal lymph nodes and lung nodules.

Given the echocardiographic findings and elevated levels of N-terminal pro–brain natriuretic peptide (1,430 pg/mL), the patient was treated with guideline-directed medical therapy for heart failure with reduced LVEF.^5^ Angiotensin-converting enzyme blood level was in the normal range. As next step, the patient underwent 2-[^18^F]-fluorodeoxyglucose positron emission tomography (PET)/CT, after standardized preparation, that included a carbohydrate-free diet in the 24 hours before and complete fasting in the 6 hours before the examination. PET/CT revealed multiple foci of intense metabolic activity with an elevated degree of radionuclide uptake in the mediastinal lymph nodes, hilum of the lungs, and pulmonary nodules, along with diffuse and patchy uptake at a myocardial level, particularly involving the IVS, inferior LV wall, and right ventricular free wall (Figure 2, A).

**Figure 3:**
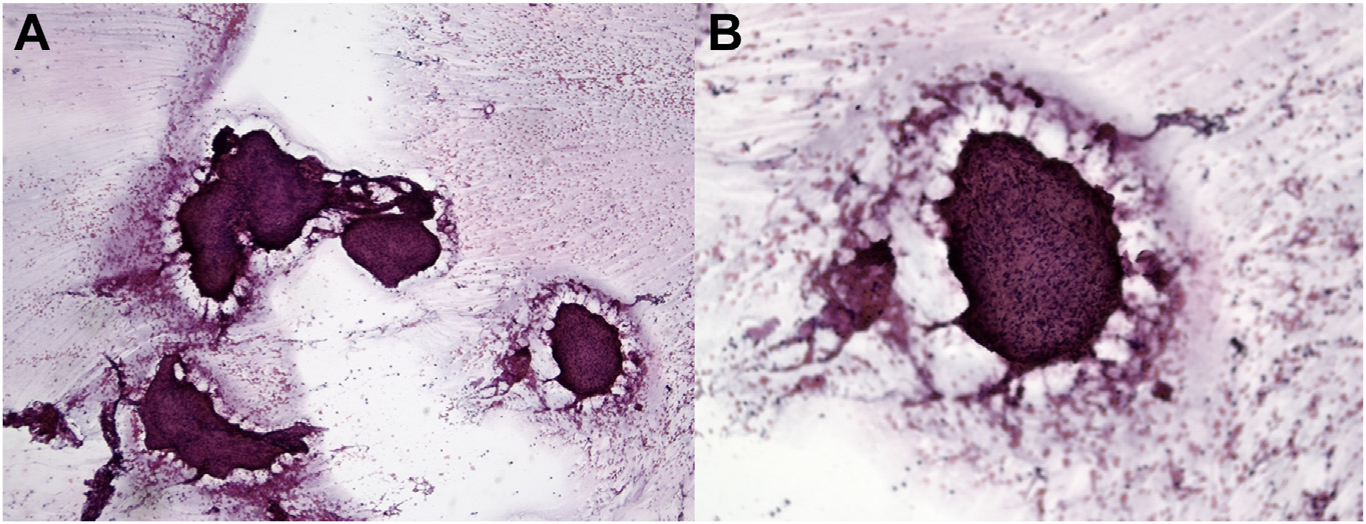
Histopathologic specimen of a mediastinal lymph node biopsy demonstrates several sharp-edged granulomas at low magnification (**A**; Papanicolaou stain, 100×) and at high magnification (**B**; Papanicolaou stain, 200×) without background necrosis.

Thus, the diagnosis of cardiac sarcoidosis was confirmed, according to international diagnostic criteria,^5^ after lymph node biopsy that showed several well-circumscribed non-necrotizing granulomas (Figure 3, A and B). Consequently, the patient started immunosuppressive therapy with prednisone and methotrexate, and after 4 months of treatment, repeat PET/CT was performed, which demonstrated a partial metabolic response at the cardiac level with minimal residual radionuclide uptake in the inferior basal LV wall (maximum standardized uptake value 7 vs 18) and complete metabolic response of the lymph nodes and lung nodules previously described (Figure 2, B). Another echocardiographic examination was performed, with only a modest improvement in LVEF to 36% (biplane Simpson method).

Nevertheless, some weeks later, the patient experienced a new episode of sustained ventricular tachycardia, interrupted by a shock delivered by the S-ICD. The patient was admitted to the hospital and underwent an electrophysiologic study, which revealed an arrhythmogenic circuit located in the thinned portion of the IVS, which has been targeted with stereotactic radioablation therapy.

**Figure 4:**
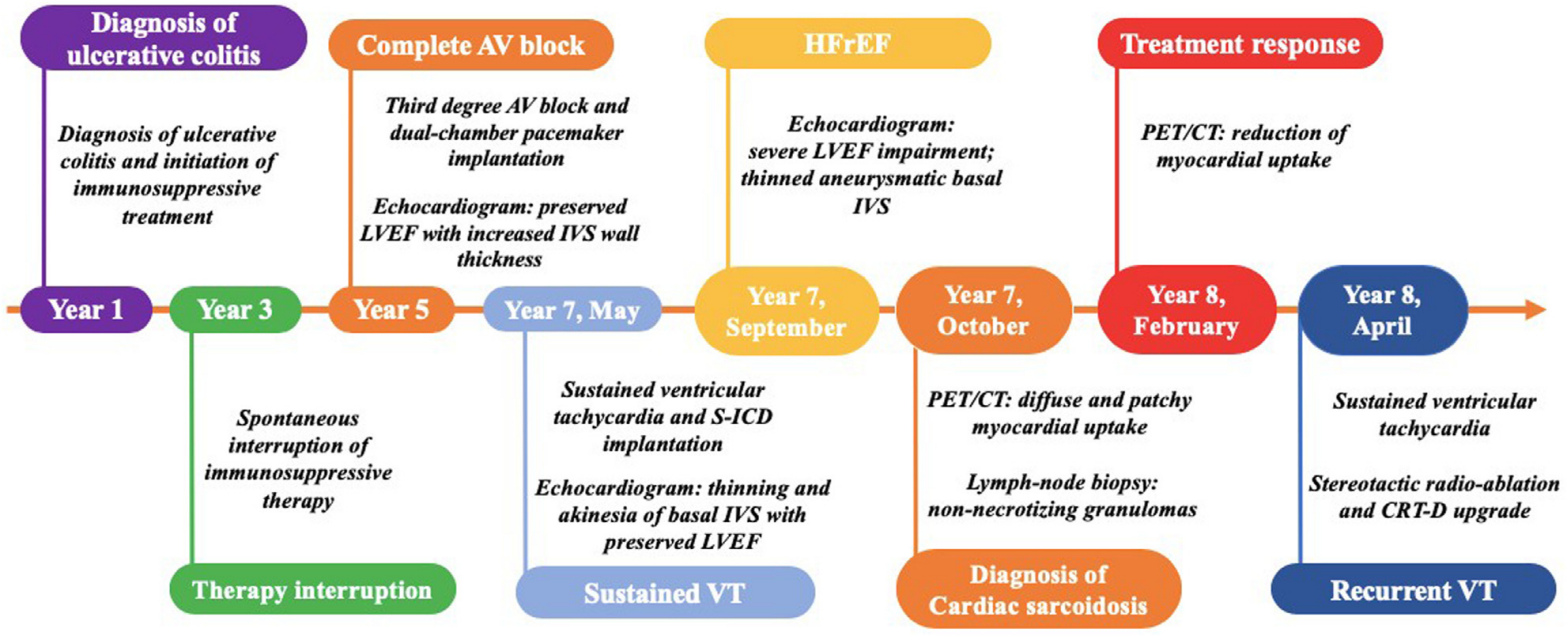
Timeline of clinical history and cardiovascular imaging findings. *CRT*-*D*, Cardiac resynchronization therapy defibrillator; *VT*, ventricular tachycardia.

Moreover, in consideration of the persistent LVEF impairment and the need for a high burden of pacing because of the complete AV block, according to current guidelines,^5^ an upgrade of the previously implanted pacemaker to a cardiac resynchronization therapy defibrillator was performed, contextually removing the S-ICD. However, the response in terms of symptomatic status, cardiac biomarkers, and LV remodeling after 6 months was modest. A timeline of the patient’ history is displayed in Figure 4.

## Discussion

We report a rare case of coexisting cardiac sarcoidosis and ulcerative colitis, highlighting the potential pathogenetic link between the two diseases and clinical implications.

Sarcoidosis is an immune-mediated granulomatous disorder that may affect multiple organs, including the heart. Cardiac involvement becomes clinically evident in 5% of patients with systemic sarcoidosis.^2^ However, the use of advanced imaging techniques enables the detection of a subclinical cardiac involvement in up to 25% of cases.^6^^,^^7^ Importantly, cardiac involvement represents one of the leading causes of morbidity and mortality in these patients, mainly related to heart failure and ventricular arrhythmias.^6^

Ulcerative colitis is an inflammatory bowel disease, and it is associated in approximately 27% of patients with extraintestinal immune-mediated manifestations, such as primary sclerosing cholangitis or ankylosing spondylitis.^3^ It has been reported that patients with ulcerative colitis have an increased risk for developing systemic sarcoidosis, with an odds ratio ranging from 1.7 (95% CI, 1.2-2.2) to 2.1 (95% CI, 1.2-2.2).^3^

The association between cardiac sarcoidosis and ulcerative colitis may be potentially related to the presence of common pathogenetic pathways, as both diseases are driven by an aberrant activation of the immune system to unknown antigens, determining a chronic inflammatory state with organ injury.^3^

Indeed, in patients with systemic sarcoidosis, the interaction between genetic susceptibility and environmental factors drives an anomalous immune response, which is mainly mediated by T helper type 1 (Th1) and Th17 cell activation.^8^ Particularly, granuloma formation requires a precise concatenation of events, starting from the antigen recognition process driven by antigen-presenting cells and CD4^+^ T cell differentiation toward Th1 and Th17 lymphocytes and the subsequent secretion of inflammatory cytokines (specifically interleukin [IL]–17A, IL-17F, IL-23, and IL-22 for the Th17 pathway).^8^^,^^9^

Moreover, specific allelic variants of the major histocompatibility complex class II molecules involved in antigen recognition, including both human leukocyte antigen (HLA) genes (HLA-DRB1∗14 and DRB1∗15) and non-HLA genes (butyrophilin-like 2 and annexin A11), have been associated with a higher risk for developing cardiac sarcoidosis.^10^

Similarly, recent studies on ulcerative colitis have suggested a crucial role of the Th17 cell pathway in triggering the inflammatory response in these patients, and increased levels of IL-23 and IL-17A have been identified in the cells of colonic mucosa on immunohistochemical analysis.^9^

In addition, allelic variants of HLA-DRB1 and butyrophilin-like 2 have been also associated with genetic susceptibility to develop ulcerative colitis.^11^

To this end, this evidence suggests that upregulation of the IL-23–Th17–IL-17A pathway and similar genetic predisposing factors may play a central role in the pathogenesis of both sarcoidosis and ulcerative colitis.

In clinical practice, the diagnosis of cardiac sarcoidosis is often challenging. The prevalence of the disease is thought to be underestimated, and coexistence with other immune disorders, including ulcerative colitis, is likely underreported. In this regard, one clinical case of association between the two diseases has been previously published.^12^ The onset of cardiac sarcoidosis followed a remote (10-year) history of ulcerative colitis, but at variance with the present case, the patient was on immunosuppressive therapy with mesalazine at the time of diagnosis, and the clinical course of cardiac sarcoidosis did not evolve toward major cardiac complications.^12^

The diagnostic challenges of cardiac sarcoidosis are related to a variety of factors: the heterogeneous clinical presentation, the lack of sensitive and effective biohumoral diagnostic markers, and the need for histologic confirmation of the diagnosis. Indeed, endomyocardial biopsy represents the diagnostic gold standard, but it may be inconclusive because of the typical patchy myocardial involvement. In this regard, the diagnosis may also be reached by histologic evidence of extracardiac disease, in association with clinical and imaging signs of cardiac involvement, after the exclusion of other etiologies for cardiac manifestations, as shown in the present case and reported in the current guidelines for the management of cardiac sarcoidosis.^7^^,^^13^^,^^14^

Echocardiography has an important role as a first-line diagnostic tool in patients with suspected cardiac sarcoidosis, despite relatively low sensitivity and specificity.^1^^,^^2^ Akinesia, thinning, and aneurysm of the basal IVS are the more characteristic echocardiographic features of cardiac sarcoidosis and reflect myocardial fibrosis and scarring due to granuloma infiltration.^1^^,^^7^ However, the estimated prevalence of these echocardiographic findings in cardiac sarcoidosis is only 30%.^15^ Other possible findings include LV wall thickening, especially in the mid to basal IVS, LV dilatation, and LV systolic dysfunction with focal or global hypokinesia. Valvular dysfunction secondary to involvement of papillary muscles and pericardial effusion are also uncommon manifestations of cardiac sarcoidosis.^2^^,^^13^

The challenges in reaching the diagnosis, as well as the risk for life-threatening complications, underscore the importance of suspecting cardiac sarcoidosis in patients presenting with suggestive clinical manifestations, such as unexplained advanced AV block, heart failure with reduced LVEF, or sustained ventricular arrhythmias, especially in young age or in presence of other immune-mediated disorders. Despite the presence of all these “red flags,” our patient experienced an important diagnostic and consequent therapeutic delay, and the disease progressed toward advanced heart failure and ventricular arrhythmias.

## Conclusion

This is a case report of cardiac sarcoidosis occurring in a patient with ulcerative colitis. Although uncommon, this association may be related to common genetic predisposing factors and pathogenetic pathways. Given the high morbidity and mortality rates linked to cardiac sarcoidosis, it is crucial to suspect the disease in high-risk populations, including patients with ulcerative colitis, aiming to provide a prompt diagnosis and treatment.

## Ethics Statement

The authors declare that the work described has been carried out in accordance with The Code of Ethics of the World Medical Association (Declaration of Helsinki) for experiments involving humans.

## Consent Statement

Complete written informed consent was obtained from the patient (or appropriate parent, guardian, or power of attorney) for the publication of this study and accompanying images.

## Funding Statement

The authors declare that this report did not receive any specific grant from funding agencies in the public, commercial, or not-for-profit sectors.

## Disclosure Statement

The authors report no conflict of interest.

## Supplementary Data

Video 1 Two-dimensional transthoracic echocardiography, parasternal long-axis view, demonstrates a dilated, hypokinetic LV cavity with regional myocardial thinning and scarring of the basal segment of the IVS with aneurysmal motion.

Video 2 Two-dimensional transthoracic echocardiography, parasternal short-axis view at the mitral valve level, demonstrates a dilated, hypokinetic LV cavity with regional myocardial thinning and scarring of the IVS.

Video 3 Two-dimensional transthoracic echocardiography, apical four-chamber view, demonstrates a dilated, hypokinetic LV cavity with regional myocardial thinning and scarring of the basal segment of the IVS with aneurysmal motion.

Video 4 Two-dimensional transthoracic echocardiography, apical two-chamber view, demonstrates a dilated, hypokinetic LV cavity with regional myocardial thinning and scarring of the basal inferior myocardial segment with aneurysmal motion.
